# Zero malaria: a mirage or reality for populations of sub-Saharan Africa in health transition

**DOI:** 10.1186/s12936-022-04340-1

**Published:** 2022-11-04

**Authors:** Esther Sarpong, Desmond Omane Acheampong, George Nkansah Rost Fordjour, Akwasi Anyanful, Enoch Aninagyei, Derek A. Tuoyire, Dee Blackhurst, George Boateng Kyei, Martins Ekor, Nicholas Ekow Thomford

**Affiliations:** 1 Department of Molecular Biology and Biotechnology, School Biological Sciences, College of Agriculture and Natural Sciences, University of Cape Coast, Cape Coast, Ghana; 2 Department of Biomedical Sciences, School Allied Health Sciences, College of Health and Allied Sciences, University of Cape Coast, Cape Coast, Ghana; 3 Pharmacogenomics and Genomic Medicine Group, Department of Medical Biochemistry, School of Medical Sciences, College of Health and Allied Sciences, University of Cape Coast, Cape Coast, Ghana; 4 Department of Biomedical Sciences, School of Basic and Biomedical Sciences, University of Health and Allied Sciences, Ho, Volta Region Ghana; 5 Department of Community Medicine, School of Medical Sciences, College of Health and Allied Sciences, University of Cape Coast, Cape Coast, Ghana; 6 Division of Chemical Pathology, Department of Pathology, Faculty of Health Sciences, University of Cape Town, Anzio Road, Observatory,, Cape Town, 7925 South Africa; 7 Department of Virology, College of Health Sciences, Noguchi Memorial Institute for Medical Research, University of Ghana, Legon, Accra, Ghana; 8 Department of Pharmacology, School of Medical Sciences, College of Health and Allied Sciences, University of Cape Coast, Cape Coast, Ghana; 9 Division of Human Genetics, Department of Pathology, Faculty of Health Sciences, University of Cape Town, Anzio Road, Observatory, Cape Town, 7925 South Africa

**Keywords:** Zero malaria, Sub-Saharan Africa, Health transition, Health systems

## Abstract

The global burden of malaria continues to be a significant public health concern. Despite advances made in therapeutics for malaria, there continues to be high morbidity and mortality associated with this infectious disease. Sub-Saharan Africa continues to be the most affected by the disease, but unfortunately the region is burdened with indigent health systems. With the recent increase in lifestyle diseases, the region is currently in a health transition, complicating the situation by posing a double challenge to the already ailing health sector. In answer to the continuous challenge of malaria, the African Union has started a "zero malaria starts with me” campaign that seeks to personalize malaria prevention and bring it down to the grass-root level. This review discusses the contribution of sub-Saharan Africa, whose population is in a health transition, to malaria elimination. In addition, the review explores the challenges that health systems in these countries face, that may hinder the attainment of a zero-malaria goal.

## Background

Malaria continues to plague countries and presents with variable country and population specific disease burden. The global malaria burden continues to be a significant public health concern despite advances made in therapeutics for malaria. There continues to be high morbidity and mortality associated with this infectious disease [1]. For advanced economies, significant improvements in their health systems and surveillance have helped minimize the effects of malaria, and most of them have achieved elimination [1]. A few low-middle income countries such as El Salvador, Sri Lanka, Paraguay and Guyana have also successfully eliminated malaria. Globally children and pregnant women are most severely affected, when infected with malaria [2]. The World Health Organization (WHO) has set a goal to reduce malaria incidence and mortality globally by 90% and subsequently eliminate malaria in at least 35 countries by 2030 [3]. While the significant challenges towards attaining these goals are a lack of adequate funding, poor infrastructure, and emergence of parasites resistant to available anti-malarial drugs [3], the overall effectiveness of health systems in affected countries with little resources has a vital role if these goals are to be met. For a long time, most sub-Saharan African (SSA) countries have been challenged significantly by infectious diseases, a battle most of them are losing amidst failing health systems, primarily due to the lack of political will and unavailability of resources. Recently, there has been an upsurge in chronic non-communicable diseases such as diabetes, obesity, hypertension, and cardiovascular diseases arising from unhealthy lifestyle trends [4, 5]. SSA countries, therefore, seem to be in a health transition as hitherto these countries were battling predominantly with infectious diseases, but now must address non-communicable diseases too. This increases stress on the already inadequate health systems. Globalization and urbanization greatly influence this health transition, thus presenting more challenges to already ailing health systems. With the WHO Africa region carrying the most significant proportion of the global malaria burden, accounting for 94% of the cases and deaths in 2019 [2], a malaria-free Africa seems daunting and near impossible vision to achieve.

If the worldwide pursuit of reduction in malaria is to be met, considerable strides need to be made in this region. Interestingly, only 5 of the 40 countries certified malaria-free by the WHO are in the WHO Africa Region [6]. This review examines strategies developed and implemented by SSA countries towards malaria elimination. It also explores the challenges that health systems in these countries face that may hinder the attainment of the zero-malaria goal.

### The high burden to high impact approach

The burden of malaria in SSA requires an enormous effort to eliminate. Global response to end malaria, involving WHO, United Nations International Children's Educational Fund (UNICEF), United Nations Development Programme (UNDP), and the World Bank led to the launch of the "Roll back malaria” (RBM) partnership in 1998. The RBM partnership, working together with WHO's Global Technical Strategy for Malaria 2016–2030 (GTS), aims that by 2030, the incidence and mortality rate of malaria will be reduced by 90% compared with 2015 levels. The GTS’ impact will be to have thirty-five countries certified as malaria-free and to safeguard re-establishment of malaria in all these countries within the period earmarked. In addition, other diseases, such as human immunodeficiency virus/acquired immunodeficiency syndrome (HIV/AIDS), tuberculosis, neglected tropical diseases, hepatitis, water-borne diseases, and other infectious diseases will be targeted and dealt with accordingly [2].

**Fig. 1:**
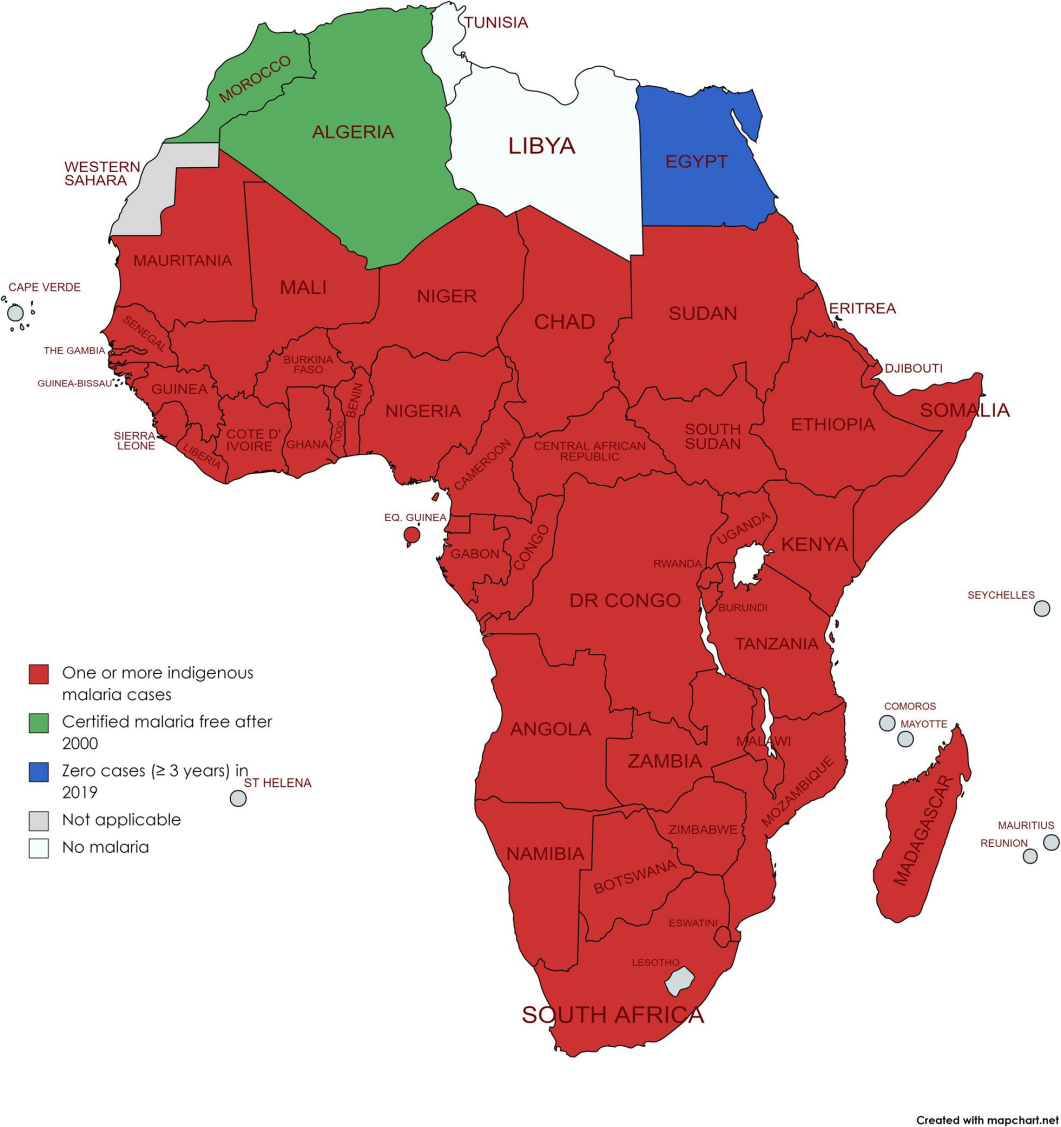
African countries with indigenous cases in 2000 and their status by 2019. Countries with zero indigenous cases over at least the past three consecutive years are considered to have eliminated malaria. (Adapted from WHO database) [1]

Good progress has been made since the 2000s, but recently, there has been an alarming plateau in progress. Most African countries are still battling malaria, though with varying prevalence and varying incidence ratios (Fig. 1). This notwithstanding, there are pockets/districts in SSA countries that have achieved local elimination or reduced disease burden. Prevalence and incidence of malaria continues to vary during the pandemic exacerbated by the impact of COVID-19 on the delivery of and access to malaria services [7–9].

In 2000, 80 cases were reported per 1000 population at risk, which reduced to 58 between 2000 and 2015, indicating a 27% malaria case incidence decline [2]. In 2019, however, the decline was marginal, at an incidence rate of 57, translating to less than a 2% decline between 2015 and 2019. This slow rate of decline, during this period, threatens the achievement of critical targets set out by the WHO and its partners [2] and has necessitated renewed efforts by countries and regional bodies.

In response to this plateau in progress, WHO implemented a new approach that targets countries that have high burden of the disease. The approach dubbed high burden to high impact (HBHI), is a country-led approach to addressing malaria [10]. HBHI hinges on four key elements. Firstly, political leaders in high burden countries together with global partners are encouraged to institute measures that are focused on translating political commitment and resources into concrete actions to eliminate malaria. Secondly, high burden countries are expected to compile and analyse strategic and quality data that relate to their peculiar needs and resources. This will ensure that interventions employed by individual countries will be tailored to answer their specific needs to create maximum impact. The third key element spells out the critical role the WHO plays as a global guide. Using the most up-to-date research, the WHO will develop global recommendations that high-burden countries can adjust for a variety of local situations and develop new instruments based on country experience. Finally, a successful approach will require a better integrated health sector response, supplemented by other sectors such as the environment, education, and agriculture [3, 10].

The success of this approach will be defined by the attainment of the GTS targets. On another level, countries are expected to increase long-term financial commitments to fighting malaria as a result of the more efficient and effective use of resources envisioned by this approach. Better malaria control will also yield demographic, social and economic benefits for each of those nations over the coming decades, all of which could be a measure of how successful the HBHI approach has been [3, 10]. Though the HBHI agenda is laudable, an outright and complete elimination of malaria in a malaria-burdened setting may seem like a tall order. Therefore, an integrated approach of reducing disease burden with a long-term goal of implementing the HBHI strategy towards elimination may seem feasible. Together with the aforementioned strategies, the four HBHI approaches and strategies if successfully implemented, would push the zero-malaria agenda to be realized.

### The zero-malaria agenda: the African version

Malaria is endemic to Africa and, therefore, it is only proper Africans develop translational campaigns and strategies that will resonate with citizens in order to eliminate malaria. The African Union's (AU) "zero malaria starts with me" is one such campaign. It is a grassroot campaign with the galvanization of community members to personalize malaria prevention to increase awareness, high on its agenda. Another critical aspect of the campaign is to encourage political prioritization of malaria elimination by all countries significantly affected, because political leaders are the custodians of government policies, decisions, and budgets. The campaign also motivates private businesses to get involved in malaria elimination and to secure additional resources and support. This campaign was first launched in Senegal in 2014, and after a successful implementation, other African countries signed on to this campaign in July 2018 and pledged their support [11, 12] to it. This means African countries are adapting the "zero malaria" agenda to their settings to realize the maximum impact. Through a country-wide strategy, this has expanded to a pan-African movement with the sole aim of inculcating political engagement, drafting the private sector, and motivating communities to become involved in the goals of the campaign. Less than a decade since the launch and adoption of the zero-malaria campaign, some African countries are adopting various practical actions and strategies to achieve impact (Table 1).

**Table 1:** Strategies employed by African countries who have launched the zero malaria starts with me campaign

Country	Initiation year	Campaign strategy
Uganda	2022	Zero Malaria Business Leadership Initiative’ to mobilize domestic financial resources Youth Engagement and National Malaria Youth Armies Establishment of an End Malaria council and Fund
Senegal	2014	Mobilization of political leaders, private sector, artiste and community members Community champions programme Free screening and treatment Use of Long-Lasting Insecticidal Nets (LLINs) ‘Zero Malaria Business Leadership Initiative’ to mobilize domestic financial resources
Mozambique	2018	End Malaria council and Fund Indoor residual spraying campaigns LLINs campaign
Zambia	2018	Establishment of an End Malaria council and Fund Mobilization of religious leaders for malaria awareness match Youth Engagement and National Malaria Youth Armies National malaria scorecards
Ghana	2019	Engaging parliamentarians through workshops and visits Boosting private sector engagement Efficient use of local domestic funds National malaria scorecards
Sierra Leone	2019	Engaging key local leaders Awareness creation by the National Malaria Control Programme Youth Engagement and National Malaria Youth Armies
Tanzania	2019	Larviciding Effective resource mobilization Fostering partnerships and accountability National malaria scorecards
Benin	2020	Zero Malaria matching fund to encourage private sector investment in malaria Zero Malaria Business Leadership Initiative’ to mobilize domestic financial resources
Kenya	2020	Distribution of mosquito nets Engaged in malaria vaccine trials Increased dissemination of public health messages Development of a network of community malaria youth champions Establishment of an End Malaria Council to attract contribution of funds from private sector National malaria scorecards
Rwanda	2020	Drones for larviciding Mass distribution of mosquito nets Indoor residual spraying campaign

Almost a decade since the launch of the “zero-malaria starts with me” agenda, only 23 countries out of the 54 African countries, have so far launched the campaign [13] in their respective countries. Considering the fact that Africa is the hardest hit when it comes to malaria, it is quite unfortunate that not a significant lot has joined the global campaign. In its 2021 report on malaria, the African Union unequivocally stated that, Africa is not on track to meet its work goal of eliminating malaria from the continent by 2030 [13] and change in disease trend within the continent further threatens the attainment of the malaria reduction targets.

Efforts are, therefore, required to sensitize Africa's population to personalize this campaign and be abreast with its health transition in order to eradicate this significant public health challenge.

### Sub-Saharan Africa's health transition: a threat to achieving zero-malaria target?

For centuries, Africa has had the burden of many infectious diseases [14]. While a number of these diseases, for example poliomyelitis, have now been eradicated (16), the continent continues to be afflicted by diseases such as malaria, HIV/AIDS, acute respiratory infections, cholera, tuberculosis, hepatitis B, Ebola, and, more recently COVID-19 [14–16] in addition to some neglected tropical diseases (NTDs), such as schistosomiasis, lymphatic filariasis, leishmaniasis, and sleeping sickness that still burden the region [17].

According to the Global Disease Report, while the disease burden of the rest of the world in 2010 seemed to be moving away from communicable diseases toward non-communicable diseases (NCDs), countries in the SSA region still had communicable diseases that affect children and young adults as the leading cause of death and disability (Tables 2 and 3).

**Table 2:** Prevalence of Non-Communicable diseases before and after the year 2000 in some Sub-Saharan African countries

Country	Disease prevalence (%)						References
	Diabetes		Hypertension		Obesity		
	Before 2000	Post 2000	Before 2000	Post 2000	Before 2000	Post 2000	
Benin					2.1	9.87	[18]
Burkina Faso					1.0	5.6	[18]
Cote d'Ivoire	< 2.0	6.83			3.0	10.95	[18, 19]
Ghana	< 2.0	6.5	16.2	23.7	3.4	11.04	[18–20]
Guinea					4.4	7.94	[21]
Mali	< 2.0	7.37			1.2	8.84	[22]
Niger					1.2	5.84	[18]
Nigeria	2.2	6.15	9.3	23.85	2.2	14.3	[23–29]
Senegal	< 2.0	7.41	10.4	24.70	3.7	8.82	[30–32]
Sierra Leone	2.0^d^ 0^e^	6.87	23.4^d^ 14.7^e^	44	5.0	8.93	[32–34]
Togo	< 2.0	7.14				8.54	[35, 36]
Central African Republic					1.1	7.64	[35, 36]
Cameroon	2.0	6.71	16.9	24.71		11.78	[35, 37]
Tanzania	0.87	6.08	9.1^a^ 12^b^	27.15	1.9	8.69	[38–40]
Ethiopia	3.70	6.50		20.6		4.62	[36, 41–43]
Uganda	< 2	4.57			1.2	5.46	[36]
South Africa	2- 4.99	11.15	14.6	26.71		28.49	[36]
Kenya	< 2	5.99			2.4	7.29	[36]
Mauritania	1.88	8.93					[36, 44]

a: men, b: women, d: urban setting, e: rural setting

**Table 3:** Prevalence of Infectious diseases before and after the year 2000 in some Sub-Saharan African countries

Country	HIV Prevalence (%)		Acute Respiratory Infection Prevalence (%)		Diarrhea Prevalence (%)		Malaria Incidence /1000 population		References
	Before 2000	Post 2000	Before 2000	Post 2000	Before 2000	Post 2000	Before 2000	Post 2000	
Benin	4.15	0.9	16	6	26	11	410.9	386.18	[45–48]
Burkina Faso	2.9	0.7	6	14	20	15	589.3	398.73	[46–48]
Cote d'Ivoire	7.5	2.1	18	8	23	16	514.61	330.73	[46–48]
Ghana	2.5	1.7	14	7	18	12	437.52	224.34	[46–48]
Guinea	2.0	6.40	17	8	22	10	458.08	283.89	[46–48]
Mali	2.0	0.9	16	6	26	15	458.08	386.78	[46–48]
Niger	0.7	0.2	15	8	39	14	317.29	356.57	[46–48]
Nigeria	1.4	1.3	12	4	16	11	438.75	291.94	[46–48]
Senegal	0.7	0.3	35	8	16	18	255.64	55.75	[46–48]
Sierra Leone	1.6	1.5	31	6	26	8	465.77	320.40	[46–48]
Togo	2.1	7.14	21	15	31	15	442.02	267.31	[46–48]
Central African Republic	7.8	2.9	45	29	26	24	458.76	347.33	[46–48]
Cameroon	4.4	3.0	20	17	19	20	386.32	246.99	[46–48]
Tanzania	6.2	4.7		35.2		12.1	342.68	124.27	[46–48]
Ethiopia	3.0	0.9		7.8	26	12	157.3	31.81	[46–48]
Somalia	0.2	0.1				22.4	125.58	34.27	[46–48]
Uganda	1.5	4.57				20	495.25	289.18	[46–48]
South Africa	10.9	19.1			13	5.3	4.23	1.65	[46–48]
Kenya	4.8	4.2		30.4		15	213.19	70.10	[46–48]
Mauritania	0.6	0.3					147.95	39.41	[46–48]

Malaria, HIV/AIDS, NTDs, diarrhoea, lower respiratory infections, meningitis, and other common infectious diseases accounted for about 35% of SSA's total disability-adjusted life years (DALYs). DALYs are a measure of the number of years of life lost due to premature death and disability [49].

In contrast, most high-income countries are currently burdened with NCDs, such as diabetes, cancers, obesity, mental illnesses, hypertension, cardiovascular diseases, lung, and kidney diseases, whilst low- and middle-income countries are still struggling with preventable communicable diseases [50].

Over the past few decades, the global distribution of disease burden has shown a change in the trend of disease landscape in African countries. Supporting data has demonstrated that chronic NCDs are rising in most of SSA [16]. Between 1990 and 2017, there was a 67% increase, for all ages, in the number of DALYs in SSA due to NCDs and a general decline in communicable, maternal, neonatal, and nutritional (CMNN) diseases [16]. This health transition, which is now paralleled by high prevalence and incidences of communicable and NCDs, is influenced practically by lifestyle changes such as tobacco and alcohol consumption and poor diet patterns [16]. Furthermore, successful implementation of treatment outcomes, such as that for anti-retroviral therapies (ARTs), has shown that the surviving population of persons living with HIV develop complications including cardiovascular diseases, cancers, renal and liver complications [51].

Urbanization in Africa, in particular, is usually swift and unplanned [9], bringing unfavourable conditions that lead to stress. These conditions include unavailability of suitable accommodation, jobs, means of transport, and even lack of healthy foods. Urbanization is linked to increased risks of unhealthy lifestyles [52–54]. Urbanization has an important effect on diet. Usually, people in urban areas rely primarily on store-bought and highly processed foods, whereas their counterparts in rural areas eat less processed foods. Such processed foods often have high fat and sugar contents and other unhealthy components that lead to lifestyle diseases [55].

Sub-Saharan Africa now has a double burden of both communicable and non-communicable diseases [16, 56]. Considering the region's health systems and policies, this poses a serious challenge to achieving the zero malaria targets.

### Healthcare systems in sub-Saharan Africa: are they adequate?

The institutions, organizations, and resources (physical, financial, and human) put together to provide health care services to satisfy the health needs of a population make up their health care system [57, 58]. The WHO defines a health system as "all organizations, people and actions whose primary intent is to promote, restore or maintain health" [59]. A robust health system would comprise a seamless integration of personal health care services, public health services, teaching and research, and health insurance. Personal health care services include primary, secondary, tertiary, and other services available at clinics, hospitals, and sometimes homes. The public health arm of a health system is usually concerned with a healthy environment, such as control of water and food supplies, regulation of drugs, and safety regulations intended to protect a given population. The teaching and research components investigate and share findings related to disease prevention, detection, and treatment whiles there is a functioning health insurance scheme to provides financial coverage for the system.

To assess the performance of health systems, the WHO instituted a six-component framework which are: (i) service delivery, (ii) health workforce, (iii) health information systems, (iv) access to essential medicines, (v) financing, and (vi) leadership/governance. A health system needs staff, funds, information, supplies, transport, communications, and overall guidance and direction to perform efficiently [60]. Therefore, to measure how good a health system is, these needs must be readily available and functional [58, 60]. For a health system to be performing, the population must enjoy essential health and health-related services where and whenever needed [60]. Unfortunately, this cannot be said about most countries in SSA because most countries within this region are low to middle-income countries confronted with several challenges in their healthcare systems [57].

When the health system performance of countries in the WHO Africa region was assessed based on the following four parameters; access to essential services, quality of essential services, effective demand by communities for basic services, and the resilience of the system to shocks, the average system performance index was 0.49 with individual countries' performance score ranging from 0.26 and 0.70. This means, on average, health systems in the region are only performing at 49% of their possible levels of functionality [60].

### Africa's health care systems: through the eyes of a daunting health transition

Poor leadership and governance are at the core of the region's health system challenges. Although most African leaders come to power with little or no experience [61], corruption among those in authority has done severe damage to the effective management of resources. The recent Corruption Perception Index (CPI) shows SSA recorded the lowest score of 33, with South Sudan having a score as low as 11 compared to high income countries such as Denmark, Finland and New Zealand that scored 88. The CPI assesses perceived public sector corruption levels by surveying experts and businesspeople in 180 countries, using a scale from 0 (highly corrupt) to 100 (very clean). A lower score indicates high corruption perception [62]. The lack of checks and balances results in a leak of public funds for development, and the health sector is not spared. Most countries in the region already have low budgetary allocation for the health sector and depend highly on donor agencies to supplement their budgets. Most countries within the region have less than 15% of the government budget allocated to health care [61, 62]. This allocation even if given in full per the country’s budget may not be enough to meet the minimum essential package of health service funding. When part of these allocations is lost due to corruption, the effect on the remaining five building blocks of a health system (service delivery, health workforce, health information systems, access to essential medicines and financing) is detrimental. A scandal that involved Ghana's health ministry in the procurement of COVID-19 vaccines is an example of how poor governance leads to lapses in health systems. Through its ministry of health, the government of Ghana paid $19 per dose for a vaccine that was selling at $10 per dose because the ministry dealt with a middleman instead of the actual producers [63].

Poor leadership trickles down from high-ranking government officials to other civil servants in various sections of the health sector. Hospital administrators, medical doctors, public health units, and other health workers are not left out. Many health workers steal drugs and other supplies, meant for hospitals, for personal gains [64, 65]. Another incident in Ghana, during the recent COVID-19 pandemics, some hospital staff were caught stealing and selling Personal Protective Equipment (PPEs) meant for use in government facilities. This was discovered by undercover journalists working for the BBC [66]. The story was no different from what happened in one of Africa’s most developed economies where there were several scandals and corruption incidences in relation to COVID 19 procurement and PPEs [67].

Treated mosquito nets and other supplies aimed at malaria prevention sometimes do not reach the populace because people in authority diverge them for personal gains. Drugs are sold to private facilities that charge individuals much higher prices than government facilities do. Individuals cannot pay and then access services that otherwise should be free or less expensive. Bad governance has a significant impact on the workforce in the sub-region. The ratio of health workers to patients in most African countries is inadequate and increases the workload on healthcare givers. Every year, the health sector in most African countries is hit by numerous industrial strikes because of dissatisfaction with working conditions and remuneration. Several groups of trained healthcare givers are unemployed because of poor governance choices and political propaganda [68–70]. A key aspect of malaria elimination is the diagnosis and treatment of patient cases [71]. A high workload on health caregivers can lead to misdiagnosis and mistreatment of patients [72].

Only a few countries like Rwanda, Ghana, Nigeria, Kenya, Uganda, and Tanzania have health insurance schemes that help to reduce the burden of medical care on individuals. Unfortunately, these are ailing schemes on the verge of collapse, and most of them are not functioning as they should [73]. This means that in most countries in this region, individuals pay cash at the point of care. The downside is that people tend not to visit hospitals when sick, and resort to self-medication. Considering the endemicity of malaria in this region, most adults may experience it countless times in their lifetime and therefore tend not to treat it urgently. People are more likely to self-medicate than go to a hospital at the onset of malaria symptoms. Failed treatment is one way by which drug resistance emerges [74]. Therefore, it is no surprise that *Plasmodium* parasites continue to develop resistance to available anti-malarial medications threatening the elimination of malaria and subsequent achievement of the zero-malaria goal in Africa.

Another dire consequence of the misappropriation of funds by leaders is that the availability of essential drugs may be affected. Circulation and use of substandard drugs are widespread in the African region. Authorities often choose to import cheaper drugs and divert funds for personal gain [75–77] Also, because most of these drugs are imported, it takes longer for them to be available because of the bureaucratic nature of the procurement and supply chain system in most countries in the region [73].

Therefore, while donor agencies and organizations tasked to work towards malaria elimination may put in place the necessary conditions and supply resources needed to ensure targets are met, the glaring lapses in most sub-Saharan African countries' health care systems pose a serious threat. If not checked efforts toward malaria elimination will be rendered ineffective. The current transition in disease burden in the region further worsens the situation and puts extra pressure on the already failing systems.

The region's seemingly poor leadership and governance are at the centre of these challenges. It becomes impossible to fix all the other issues without addressing the leadership challenge, so efforts should be directed toward instituting checks and balances within governments. To deter others, strict punitive measures should be meted out to offenders where checks and balances fail. Until the region straightens its leadership and governance problems, malaria elimination appears more of a mirage than a reality.

To achieve the zero-malaria goal, public health education regarding malaria should be paramount as most citizens in African countries are not well informed or misinformed regarding health issues, most especially with relatively "common" diseases such as malaria.

Disease trends have shown that most conditions result from interactions between humans and their environment, which is similar in malaria. Most public health education systems fail to address the vector that carries the malaria parasites in the environment. Achieving zero malaria for SSA should start with eliminating the cause of the disease, and aim to eliminate mosquitoes that carry the parasite. The community engagement of the "zero-malaria starts with me" agenda should be apt as through proper community engagement and education, most African countries may stand a chance of achieving the 2030 agenda of reducing the malaria burden by 90% and thus moving closer to eliminating malaria.

### Zero malaria: the science of optimized therapy for malaria

For regions in health transition, most African countries continue to practice medical herbalism, and orthodox medicine [78–80] due to some of the factors elucidated earlier. Herbal medicine is cheap and primarily affordable; however, not all can treat malaria. There is insufficient data on their efficacy and quality [81–83]. Misinformation influences the treatment of malaria in both health systems, leading to parasite resistance. Malaria presumptive self-diagnosis is on the ascendancy in most African countries, especially in rural communities [84–86].

When the efficacy of some commonly used herbal preparations was compared to that of WHO-recommended artemether-lumefantrine in *Plasmodium berghei* in vivo, their parasite clearance ability was lower, and they were unable to completely clear all parasites, which raises concern for drug and parasite resistance [87, 88]. Unfortunately, once treatment is initiated and symptoms disappear, patients stop treatment, building towards parasite resistance. Herbal medicines are multi-phyto constituted and the mechanism of action of these components may be synergistic or opposing which may, therefore, have variable effects on parasites and, therefore, can potentially lead to parasite drug resistance.

On the one hand is the quality of the WHO-recommended artemisinin-based combination therapy (ACT) available in SSA. A high proportion of artemisinin-based combinations sold in most SSA countries have been proven to have poor quality [89–91]. Some of the medications are inferior and may not contain the indicated amount of active pharmaceutical ingredient (API), have poor API dissolution, or have labels that make deceptive claims about content and origin and may not even include the claimed API, as well as include erroneous, undeclared chemicals [92]. The problem of poor-quality ACT is even more predominant in the private sector, where business owners stock cheaper drugs, and usually these have low quality. The effect of this is the emergence of drug-resistant strains that no longer respond to ACT.

Unfortunately, most countries within SSA do not have rigid regulations to check the influx of poor-quality ACT, which significantly threatens the much sought-after elimination of malaria. Until systems and checks are in place, countries can take advantage of the WHO prequalification of drugs and ensure that only pre-qualified medicines are allowed [92, 93]. To achieve malaria set targets, accurate diagnosis and high-quality ACT will ensure effective treatment and significant parasite clearance. Education on the use of appropriate treatment strategies at the grass-root level will also ensure that there is avoidance of practice of medical herbalism and orthodox treatment for malaria, which will potentially help reduce parasite resistance and the emergence of new strains of the parasite. Research will form a key to reducing the malaria burden in sub-Saharan Africa.

### Research as a tool for the zero-malaria agenda

The goal of malaria elimination cannot be achieved without solid research and innovation, particularly from scientists who are directly affected by the disease in their daily life. Until recently, research scientists were in short supply in malaria endemic SSA, and as such, their contribution to malaria research was minimal [94, 95].

With the establishment of initiatives such as the Malaria Capacity Development Consortium (MCDC) [94], Multilateral Initiative on Malaria (MIM) [96], African Malaria Network Trust (AMANET), West Africa Network for Clinical Trials for Antimalarial Drugs (WANECAM), African Media and Malaria Research Network (AMMREN), West Africa Malaria Initiative (WAMI), Malaria Research and Training Center (MRTC) and Anti-malarial Drug Resistance Network (ADRN), there are renewed efforts to collaborate, share ideas and train young scientist to contribute to research in malaria.

SSA still needs adequate investment in basic science and implementation research and innovation to create new tools and strategies to combat the ever-evolving parasites [97]. The contributions of African governments and the private sector toward malaria research funding are overwhelmingly low [13, 98]. African universities and research institutes should place malaria research as top of their priorities. Evidence suggests there is unequal research investment across SSA, with countries such as Tanzania, Kenya, and Uganda receiving relatively significant investments in malaria-related research. In contrast, others like the Central African Republic and Sierra Leone receive little to no investments [99].

Key areas of research that need critical attention are the development of new therapeutic options and diagnostics, systematic operational research, vector control methods, and vaccine development [100]. There is a need for national operational research to also evaluate the operational feasibility, safety, and cost-effectiveness of WHO-recommended tools and strategies and tailor them to suit their environment and needs.

## Conclusion

A look at the health systems, campaign strategies and reluctance to even join the zero-malaria agenda shows with a less than a decade to get to the proposed time frame of achieving 90% malaria reduction in malaria endemic zone, zero malaria targets in SSA may not be achieved rapidly, but it is possible, provided the necessary changes that would ensure it, are enforced. The AU's "zero malaria starts with me campaign" is a step in the right direction. The campaign can mobilize all the sectors that have critical roles to end malaria, from political leaders to community members who are the most affected by the disease. Malaria can be reduced to the barest minimum, if not eradicated, with combined efforts from political leaders, the private sector, and community members.

## Abbreviations

WHO: World Health Organization
SSA: Sub-Saharan African
UNICEF: United Nations International Children's Educational Fund)
UNDP: United Nations Development Programme
RBM: Roll back malaria
GTS: Global Technical Strategy for Malaria
HIV/AIDS: Human Immunodeficiency Virus/ Acquired Immunodeficiency Syndrome
HBHI: High burden to high impact
AU: African Union's COVID-19
NTDs: Neglected Tropical Diseases
NCDs: Non-Communicable Diseases
DALYs: Disability-Adjusted Life Years
CMNN: Communicable, maternal, neonatal, and nutritional
ARTs: Anti-retroviral therapies
CPI: Corruption Perception Index
PPES: Personal Protective Equipment
API: Active pharmaceutical ingredient
MCDC: Malaria Capacity Development Consortium
MIM: Multilateral Initiative on Malaria
AMANET: African Malaria Network Trust
WANECAM: West Africa Network for Clinical Trials for Antimalarial Drugs
AMMREN: African Media and Malaria Research Network
WAMI: West Africa Malaria Initiative
MRTC: Malaria Research and Training Center
ADRN: Anti-malarial Drug Resistance Network
